# Rapid and sensitive identification of *Candida* in blood based on M1 beads enrichment combined with multiple recombinase-aided PCR: a culture-independent approach

**DOI:** 10.3389/fcimb.2025.1552529

**Published:** 2025-03-13

**Authors:** Yuxin Wang, Xiaoping Chen, Kenan Peng, Yanqing Tie, Yuan Gao, Zhiqiang Han, Xiaona Lyu, Hongyi Li, Ruiqing Zhang, Shijue Gao, Xinxin Shen, Xuejun Ma, Zhishan Feng

**Affiliations:** ^1^ Graduate School, Hebei Medical University, Shijiazhuang, Hebei Province, China; ^2^ Department of Clinical Laboratory, Hebei General Hospital, Shijiazhuang, Hebei Province, China; ^3^ National Key Laboratory of Intelligent Tracking and Forecasting for Infectious Diseases, National Institute for Viral Disease Control and Prevention, Chinese Center for Disease Control and Prevention, Beijing, China; ^4^ National Institute for Communicable Disease Control and Prevention, Chinese Center for Disease Control and Prevention, Beijing, China; ^5^ Hebei Key Laboratory of Molecular Medicine, Shijiazhuang, Hebei Province, China; ^6^ Hebei Clinical Research Center for Laboratory Medicine, Shijiazhuang, Hebei Province, China; ^7^ Graduate School, Hebei North University, Zhangjiakou, Hebei Province, China

**Keywords:** *Candida*, bloodstream infection, recombinase-aided PCR assay(RAP), recombinant human mannan-binding lectin protein beads (M1 beads), multiple detection

## Abstract

**Introduction:**

Clinically, timely diagnosis and effective treatment of *Candida* bloodstream infections rely on rapid and sensitive detection methods. However, the long turn-around time and low detection rate of blood culture (the gold standard) make rapid diagnosis of *Candida* challenging. This study develops a novel molecular assay (M1-mRAP) designed for the rapid and sensitive detection of three *Candida* species in blood samples: *Candida albicans*(CA), *Candida tropicalis*(CT), and *Candida glabrata*(CG).

**Methods:**

We used the M1-mRAP method aimed at detecting *Candida* DNA in blood samples, in which we developed a novel multiplex recombinase-aided PCR (mRAP) assay for sensitive amplification of *Candida* DNA and used a self-developed recombinant human mannan-binding lectin beads (M1 beads)method for enrichment of *Candida* in blood. The analytical sensitivity of mRAP was evaluated using *Candida* recombinant plasmids. The analytical sensitivity of the M1-mRAP method for blood sample detection was assessed using quantitative *Candida* simulated blood samples. The clinical performance of the mRAP and M1-mRAP methods was evaluated in 120 non-blood samples and 9 blood samples and compared with conventional qPCR methods.

**Results:**

The limit of detection(LOD) for CA, CT, and CG by the mRAP method were 4, 4, and 3 copies/μL, respectively. The LOD for CA, CT, and CG simulated blood samples by the M1-mRAP were 2, 2, and 1 CFU/mL, and the overall detection time was about 3.5 h. Clinical assays of mRAP and M1-mRAP showed that these two methods were consistent with qPCR (P<0.05), but had better clinical detection ability than qPCR. Specifically, the mRAP method identified 5 (4.2%) qPCR-negative samples, while M1-mRAP detected 1 (11.1%) classified as the qPCR grey zone sample.

**Conclusion:**

The M1-mRAP method provides rapid and sensitive detection of low concentrations of CA, CT, and CG blood samples and has the potential to emerge as an important tool for the early detection of *Candida* bloodstream infections in clinical settings.

## 1 Introduction

Bloodstream infections (BSIs), associated with high mortality rates and high healthcare costs, have become a health priority issue worldwide (Reinhart et al., 2017). Among these, *Candida* bloodstream infections are particularly notable, ranking as the fourth most common cause of hospital-associated BSIs and the most common cause of fungal infections in hospitalized patients (Kullberg and Arendrup, 2016). Recent statistics indicate that approximately 1,565,000 individuals develop *Candida* bloodstream infections or invasive candidiasis each year, with an estimated 995,000 of these cases leading to death—an alarming 63.5% mortality rate (Denning, 2024). Compounding this issue is the rising problem of *Candida* drug resistance. Frequent and prophylactic clinical use of antifungal drugs has led to strong resistance in many *Candida* species, particularly *Candida albicans*, *Candida glabrata*, and *Candida auris* (Brescini et al., 2020; Lee et al., 2021). This trend presents a major clinical challenge in treating *Candida* infections, as limited antifungal drugs are available for systemic therapy (Fang et al., 2023). Given this landscape, timely and accurate identification of the specific *Candida* infection is necessary for efficient clinical management to allow timely administration of the appropriate antifungal medication. Currently, the gold standard for clinical microbiological detection of *Candida* bloodstream infections is blood culture (Pappas et al., 2018). Although blood cultures have good analytical sensitivity with a limit of detection(LOD) of 1-10 CFU/mL, the drawbacks are obvious, mainly the low detection rate (about 70%) and the long turn-around time (Pfeiffer et al., 2011; Pappas et al., 2018; Ransom et al., 2021). The concern is that these limitations may put patients at higher risk by causing delays in antifungal drug therapy (Pruinelli et al., 2018). The importance of prompt treatment is underscored by research indicating that delayed antibiotic administration within 6 hours of identification of patients with severe sepsis and septic shock is significantly associated with increased mortality (Ferrer et al., 2014). In contrast, blood culture methods are inadequate for early clinical diagnosis, emphasizing the need and importance of non-culture methods (Ganguli et al., 2022).

In *Candida* non-culture assays, quantitative Polymerase Chain Reaction (qPCR) assays for nucleic acid detection may be the best option due to their higher sensitivity and faster speed. Among commercially available qPCR systems, the only FDA-approved T2 *Candida* Panel system (T2MR) exhibits considerably superior performance. T2MR based on PCR and magnetic resonance technologies to rapidly identify the five most common *Candida* species: *Candida albicans* (CA), *Candida tropicalis* (CT), *Candida glabrata* (CG), *Candida krusei* (CK), and *Candida parapsilosis*(CP), directly from whole blood in less than 5 hours, with a LOD as low as 1-3 CFU/mL (Pfaller et al., 2016; Zervou et al., 2017; Monday et al., 2021). Despite its excellent performance characteristics, the necessary supporting equipment and the limited number of *Candida* species hinder the widespread citation of T2MR in primary care settings. Other commercially available *Candida* qPCR assays typically utilize conventional multiplex qPCR methods, and most rely on blood cultures as the first detection step (Ganguli et al., 2022). A few qPCR assays can detect nucleic acids directly from whole blood or serum but have low overall detection limits (Ganguli et al., 2022). Simply put, nonculture methods for *Candida* that can be widely used in clinical practice are still lacking. Consequently, there is a pressing need for new, simple, rapid, and sensitive *Candida* blood tests to enhance early diagnosis and improve patient outcomes.

Our group previously reported a novel recombinase-aided PCR (RAP) method based on recombinant human mannan-binding lectin(rhMBL;i.e., M1 protein) enrichment for the detection of *Candida* in blood samples from patients with *Candida* BSIs and a dual-RAP detection of CA and CT at low concentrations (<10 CFU/mL) in blood in approximately 4-5 hours (Chen et al., 2020; Zhang et al., 2023). The RAP method combined recombinase-aided amplification (RAA) and qPCR and allowed a two-system reaction in one tube by Docosane separation (Fan et al., 2023). However, the system has some limitations. Firstly, the RAA and qPCR systems are separated by heating meltable Docosane, resulting in a highly cumbersome preparation process. Secondly, detecting each target in the RAP reaction requires two pairs of primers (one pair each for RAA and qPCR), complicating primer design and screening. Additionally, the total detection time of 4-5 hours is longer than expected and could be further optimized.

In this study, we aimed to address the limitations of the reported dual-RAP method for *Candida* detection by developing a single-system multiplex RAP (mRAP) reaction based on M1 beads enrichment, referred to as M1-mRAP. The M1-mRAP method consists of two steps: M1 enrichment and nucleic acid amplification. The M1 enrichment aims to increase the concentration of *Candida* in the blood, while the nucleic acid amplification ensures the specificity, rapidity, and sensitivity of the reaction. The method was designed to detect three common clinical *Candida* species in China: CA, CT, and CG. The single-system mRAP employs only one pair of RAA primers for each target and integrates the RAA and qPCR systems into a Docosane-free single-system reaction, providing a simpler reaction system and faster detection time. The performance of the M1-mRAP was further validated using simulated quantitative blood samples and clinical samples to demonstrate its efficacy for rapid and accurate detection of three *Candida* species.

## 2 Materials and methods

### 2.1 Sample collection and nucleic acid extraction

The standard strains of CA (ATCC 753), CT (ATCC 750), CG (ATCC 2001), and M1 protein were provided by the Institute of Infectious Diseases, Chinese Center for Disease Control and Prevention(China CDC). A total of 120 non-blood clinical samples (30 each of CA, CT, and CG blood culture-positive and 30 blood culture-negative) were provided by the microbiology laboratory of the clinical laboratory of Hebei General Hospital, from April 26, 2024, to September 23, 2024. Sample types included sputum, urine, bile, and alveolar lavage fluid. Clinical samples were collected and transported on ice to the central laboratory of the Institute of Virology of the China CDC and stored in a -80°C refrigerator. Nine fresh clinical blood samples were provided by the Hematology Department of Peking University First Hospital from October 11, 2023, to August 5, 2024. Fresh blood from patients with suspected fungal infections was collected at the hospital, placed in heparin anticoagulation tubes, and kept on ice for immediate transport (within 2 hours) to the central laboratory. M1 beads enrichment and nucleic acid extraction steps were performed immediately in the laboratory to ensure *Candida* cell activity. In addition, simultaneous sample processing in the laboratory and sample sequencing analysis or blood culture testing in the hospital were performed. Nucleic acids of the strains were extracted using FastPure^®^ Microbiome DNA Isolation Kit (Vazyme, Nanjing, China) according to the instructions.

### 2.2 Design for single-system mRAP primer and probe and construction of plasmids

The complete ITS gene sequences of CA, CT, and CG were downloaded from the National Center for Biotechnology Information(NCBI) and were aligned using Bioedit software to identify highly conserved regions suitable for plasmid design. A pair of RAA primers and a TaqMan probe were required for each target in the single-system mRAP reaction. The RAA primers of CA, CT, and CG were designed using Primer 6 and AmplifX software according to RAA primer design principles, and their effectiveness was further validated using Primer-BLAST on the NCBI website. The TaqMan probes for CA and CT were sourced from a previous study in our laboratory (Zhang et al., 2023), while the CG probe was obtained from the literature (Metwally et al., 2007). The sequence information for the single-system mRAP is shown in **Table 1**. All primers and probes were synthesized and purified by Sangon Biotech(Shanghai, China). The conserved ITS gene fragments of CA, CT, and CG were cloned into the pUC57 vector to synthesize recombinant plasmids, respectively (TsingKe Biotech, Beijing, China). The concentration of the recombinant plasmids was quantified using the Qubit^®^ dsDNA HS Assay Kit (Thermo Fisher Scientific, MA, USA) in conjunction with the Qubit 2.0 Fluorescence Quantifier (Life Technologies, California, USA). The recombinant plasmid copy numbers were calculated as follows: plasmid copy number (copy number/μL)=[6.02×10^23^×plasmid concentration (ng/μL)×10^-9^]/[plasmid length×660] (Li et al., 2023). The plasmids were diluted gradient from 10^8^ ~ 10^0^ copies/μL using TE buffer and stored at -20°C for backup.

**Table 1:** Primer and probe sequences used in mRAP.

Candida	Primer/probe	Sequence(5’-3’)	Source
CA	CA-RAA-F	TTGGGTTTGCTTGAAAGACGGTAGTGGTAA	This study
	CA-RAA-R	TCCTCCGCTTATTGATATGCTTAAGTTCAG	This study
	CA-qPCR-F	GCTTGAAAGACGGTAGTGGT	This study
	CA-qPCR-R	GTAGTCCTACCTGATTTGAG	This study
	CA-qPCR-P^a^	FAM-ATTGCTTGCGGCGGTAACGTCC-BHQ1	(Zhang et al., 2023)
CT	CT-RAA-F	GTTGAGCAATACGCTAGGTTTGTTTGAAAG	This study
	CT-RAA-R	TCCTCCGCTTATTGATATGCTTAAGTTCAG	This study
	CT-qPCR-F	TAAGCGACTTAGGTTTATCC	This study
	CT-qPCR-R	GTAGTCCTACCTGATTTGAG	This study
	CT-qPCR-P^a^	CY5-AACGCTTATTTTGCTAGTGGCC-BHQ3	(Zhang et al., 2023)
CG	CG-RAA-F	AGCTTCTCTATTAATCTGCTGCTCGTTTGC	This study
	CG-RAA-R	TGATTTGAGGTCAAACTTAAAGACGTCTGT	This study
	CG-qPCR-F	CTATTAATCTGCTGCTCGTTT	This study
	CG-qPCR-R	ACGCACAAAACACTCACTTA	This study
	CG-qPCR-P^a^	HEX-TAGGTTTTACCAACTCGGTGTTGAT-BHQ1	(Metwally et al., 2007)

a Probe modifications: FAM, 6-carboxyfluorescein; HEX, 6-hexachlorofluorescein; CY5, Cyanine 5; BHQ, black hole quencher; CA, *Candida albicans*; CT, *Candida tropicalis*; CG, *Candida glabrata*.

### 2.3 Establishment and optimization of single-system mRAP

#### 2.3.1 Two-system mRAP assay

**Figure 1:**
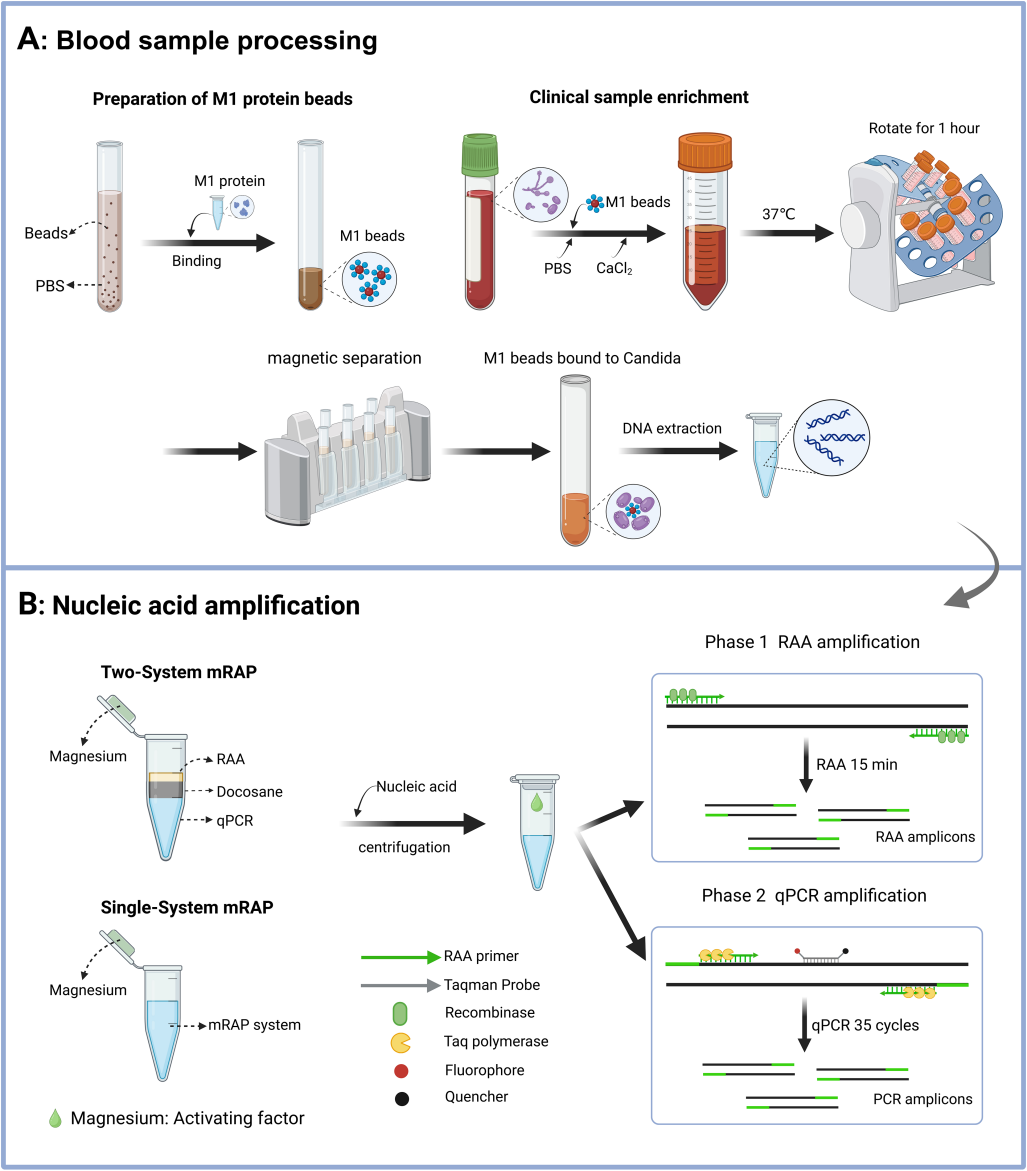
Schematic diagram of the M1-mRAP method. The process of M1 beads enrichment of blood samples includes the preparation of M1 beads and the enrichment of blood samples **(A)**; the two-system mRAP method and the single-system mRAP method possess different reaction systems but the same detection principle, RAP **(B)**. M1 protein, recombinant human mannan-binding lectin protein; M1 beads, Magnetic beads coated with M1 protein; RAP, recombinase-aided PCR. This figure was created by Biorender.

Before optimizing the mRAP system, a two-system mRAP assay for the detection of CA, CT, and CG was established based on previous research and optimization in our laboratory (Zhang et al., 2023). As shown in **Figure 1**, the RAA system (top layer) and the qPCR system (bottom layer) in the two-system mRAP are located in two separate spaces of the same tube, separated by heating meltable Docosane (middle layer) (JSNEB, Hong Kong, China). The first stage was carried out at 39°C, with Docosane remaining in a solid state (melting point 45°C) and the RAA reacting normally to rapidly amplify a large amount of template DNA within 15 minutes. Subsequently, the temperature was increased to 95°C, causing the Docosane to melt to a liquid state and rise to the top surface because of its lower density than the RAA mixture. This change caused the RAA reaction product to fall to the bottom layer of the qPCR system, initiating the second stage. In the second stage, the RAA reaction product served as a template for the qPCR reaction, and the qPCR reaction was performed. In this stage, the enzymes in the RAA system were inactivated at high temperature without affecting the normal qPCR reaction. A pair of RAA primers and a pair of PCR primers were used for each target in the two-system mRAP. The RAA primers used in two-system mRAP were consistent with the single-system mRAP, and the PCR primers were optimized after screening. The primer sequences are shown in **Table 1**.

The total reaction volume of the two-system mRAP assay was 50 µl. The upper RAA reaction system (10 µl) contained a DNA template, 14 mM of magnesium acetate, 0.2 μM of RAA primer, and RAA buffer (Amp-Future, Changzhou, China). The bottom qPCR reaction system (40 μl) contained 250 nM of each qPCR primer, 250 nM of each probe, 150 μM of dNTP, 2.5 U of Taq enzyme, 1.5 mM of MgCl2, and 4× qPCR buffer (ABclonal, Wuhan, China). The amplification procedure was as follows: 39°C for 15 min, 95°C for 3 min (1 cycle, RAA reaction and enzyme inactivation), 95°C for 15 s, and 60°C for 45 s (35 cycles, qPCR reaction). Nuclease-free water was used as a negative control, and standard strain nucleic acids served as a positive control throughout the assay.

#### 2.3.2 Single-system mRAP assay

Single-system mRAP is based on the same principle as two-system mRAP, the only difference being that the single-system mRAP removes the Docosane and integrates the two systems into one reaction system. In this single system, the RAA reaction is first performed at 40°C to amplify a large amount of template. The temperature was then raised to 97°C to inactivate the enzyme used in the RAA reaction, and the resulting RAA product was used as the PCR template for the qPCR reaction. Advantages of single-system mRAP include a simpler reaction system, easier operation, and high sensitivity (comparable to two-system mRAP). Several parameters were carefully adjusted to optimize the single-system mRAP, including the composition of the buffer, the ratio of primers to probes, the selection of qPCR enzymes, and the concentration of magnesium ions.

The final composition of the single-system mRAP reaction system (20 µl) consisted of 7 mM of magnesium acetate, 200 nM of each RAA primer, 266 nM each of CA-P and CG-P, 200 nM of CT-P, Taq enzyme 2U (TOYOBO, Japan), 20 mM of betaine (Sigma-Aldrich, USA), and RAA buffer (Amp-Future, Changzhou, China). After nucleic acids were added to the tubes, the magnesium acetate (Reaction initiation factor) was released from the centrifuge cap into the reaction mixture to initiate the RAA reaction. The amplification procedure was as follows: 40°C for 15 min, 97°C for 2 min (1 cycle, RAA reaction and enzyme inactivation), 97°C for 15 s, and 60°C for 1 min (35 cycles, qPCR reaction). The principle of the single-system mRAP is shown in **Figure 1**. Nuclease-free water was used as a negative control, and standard strain nucleic acid served as a positive control throughout the assay.

### 2.4 Specificity, sensitivity and reproducibility of single-system mRAP assays

The sensitivity of the single-system mRAP method was simultaneously evaluated by single-system mRAP, two-system mRAP, and qPCR using CA, CT, and CG recombinant plasmids at concentrations of 10^5^-10^0^. The reproducibility of the single-system mRAP methods was evaluated by testing each concentration eight times, each test using nuclease-free water as the negative control.

Considering the practical application in clinical settings, specificity assay was performed in the bloodstream environment. Fifteen common pathogens associated with bloodstream infections were selected, including *Candida albicans*, *Candida tropicalis*, *Candida glabrata*, *Candida krusei*, *Candida parapsilosis*, *Aspergillus flavus*, *Staphylococcus aureus*, *Klebsiella pneumoniae*, *Streptococcus pneumoniae*, *Pseudomonas aeruginosa*, *Escherichia coli*, *Pseudomonas maltophilia*, *Enterococcus faecalis*, *Enterobacter cloacae*, and *Mycobacterium tuberculosis* (**Table 2**). First, the 15 pathogens were passaged and cultured. After growth, they were added to 400 μL of blood from healthy individuals, followed by manual extraction of nucleic acids using the FastPure^®^ Microbiome DNA Isolation Kit (Vazyme, Nanjing, China). Finally, the extracted nucleic acids were assayed using the triple mRAP.

**Table 2:** Microbial strains used in specificity assay.

Strains	Origin	CA	CT	CG
*Candida albicans*	ATCC 753	Positive	Negative	Negative
*Candida tropicalis*	ATCC 750	Negative	Positive	Negative
*Candida glabrata*	ATCC 2001	Negative	Negative	Positive
*Candida krusei*	ATCC 6258	Negative	Negative	Negative
*Candida parapsilosis*	ATCC 22019	Negative	Negative	Negative
*Aspergillus flavus*	Isolated strains	Negative	Negative	Negative
*Staphylococcus aureus*	ATCC 29213	Negative	Negative	Negative
*Klebsiella pneumoniae*	ATCC 11296	Negative	Negative	Negative
*Pseudomonas aeruginosa*	ATCC29213	Negative	Negative	Negative
*Streptococcus pneumoniae*	Isolated strains	Negative	Negative	Negative
*Escherichia coli*	Isolated strains	Negative	Negative	Negative
*Pseudomonas maltophilia*	Isolated strains	Negative	Negative	Negative
*Enterococcus faecalis*	Isolated strains	Negative	Negative	Negative
*Enterobacter cloacae*	Isolated strains	Negative	Negative	Negative
*Mycobacterium tuberculosis*	Isolated strains	Negative	Negative	Negative

CA, *Candida albicans*; CT, *Candida tropicalis*; CG, *Candida glabrata*.

### 2.5 Preparation of quantitative simulated blood specimens

Quantitative simulated blood samples of CA, CT, and CG were prepared following a protocol described below and used to determine the LOD of single-system mRAP and M1-mRAP methods in blood samples. To begin, the standard strains of CA, CT, and CG, which had been preserved at -80°C, were thawed and inoculated into Sabouraud’s medium. These inoculated strains were then placed in a CO_2_ incubator (Thermo Fisher Scientific, USA) at 37°C for 24 to 48 hours to allow for fungal growth. After the incubation period, single colonies of *Candida* were selected from Sabouraud’s medium and further inoculated into the YPD medium. This culture was incubated overnight at 25°C with shaking at 190 rpm (JMLABO, Germany) to ensure adequate growth. The following day, 1 mL of the cultured *Candida* suspension was collected and washed three times with phosphate-buffered saline (PBS), and the concentration of the *Candida* cells was determined using a Neubauer counting chamber. The prepared *Candida* suspensions were then diluted with PBS and added to 1 mL of human blood to create simulated blood samples with final concentrations: <10, 20, 50, 100, 200, 300, 500, and 1000 CFU/mL (CFU, colony-forming units). Concurrently, PBS samples at the same concentrations were also prepared and incubated in Sabouraud’s medium at 37°C to accurately calculate the actual CFU concentrations in the simulated blood samples.

### 2.6 M1 enrichment of blood samples

#### 2.6.1 Preparation of M1 beads complex

M1 beads complex were prepared for subsequent M1 enrichment steps following the procedure. First, 500 µl of magnetic beads (5 mg) were placed into a 14 ml tube, washed three times with 5 mL PBS buffer (1×), and magnetically separated after each wash. Subsequently, 1250 µl of M1 protein (1 mg/mL) and 6.5 ml of PBS buffer were added to the tube, gently blown and mixed, and suspended on a rotating rack at low speed for 30 minutes at room temperature to facilitate the formation of the M1 beads complex. After the incubation period, magnetic separation was performed using a magnetic rack for 5 minutes to isolate the M1 beads complex. Finally, the M1 beads were stored at 4°C until use (validity period within 2 weeks).

#### 2.6.2 M1 enrichment and nucleic acid extraction from simulated and clinical blood samples

Begin by adding 1-3 mL of fresh blood, whether a low-concentration simulated blood sample (1-10 CFU/mL) or a clinical blood sample, into a 14 mL round bottom tube. Next, the total volume of the system was adjusted to 10 mL by adding PBS. Following this, 100 μL of M1 beads and 40 μL of 1 M CaCl_2_ were incorporated into the tube. Subsequently, the lid was securely sealed with a sealing film, and the tube was placed on a rotating rack at 37°C for 55 minutes at a low speed. After the suspension period, magnetic separation was conducted for 8 minutes using a magnetic rack. Then, 400 µl of PBS buffer was added to resuspend the M1 beads. DNA extraction was performed on the 400 µl of clinical samples or enrichment fluid using a nucleic acid extraction kit (Vazyme Biotech, Nanjing, China). The extracted nucleic acids were eluted with 50 µl of eluent, stored in 1.5 ml EP tubes, and stored at -80°C until use. Additionally, a second blood simulation sample of the same concentration underwent identical enrichment procedures and was transferred to Sabouraud’s medium for overnight incubation. This step was intended to calculate the actual CFU concentration of *Candida* in the enriched blood sample.

### 2.7 Detection evaluation of simulated samples and clinical samples

Nucleic acids from CA, CT, and CG high-concentration simulated blood samples (20-1000 CFU/mL) were tested by single-system mRAP and qPCR and compared, to evaluate the performance of the single-system mRAP assay for the detection of blood specimens. After M1 beads enrichment and nucleic acid extraction of CA, CT, and CG low-concentration blood simulated samples (1-10 CFU/mL), single-system mRAP and qPCR were performed and compared, to evaluate the performance of the M1-mRAP assay for the detection of blood specimens. Nucleic acids from 120 non-blood clinical samples were tested by mRAP and qPCR and compared to evaluate the performance of the single-system mRAP assay for non-blood clinical samples. Nine fresh blood samples were enriched with M1 beads, and the extracted nucleic acids were tested by single-system mRAP and qPCR and compared to evaluate the performance of the M1-single-system mRAP assay for clinical blood samples.

### 2.8 Statistical analysis

Probit analysis was employed to determine the 95% probability detection limit of single-system mRAP. The detection results were analyzed using the Kappa test, with p-values below 0.05 deemed statistically significant. All statistical analyses were performed using SPSS 26.0 (IBM) software.

## 3 Results

### 3.1 Optimization of the single-system mRAP assay

**Figure 2:**
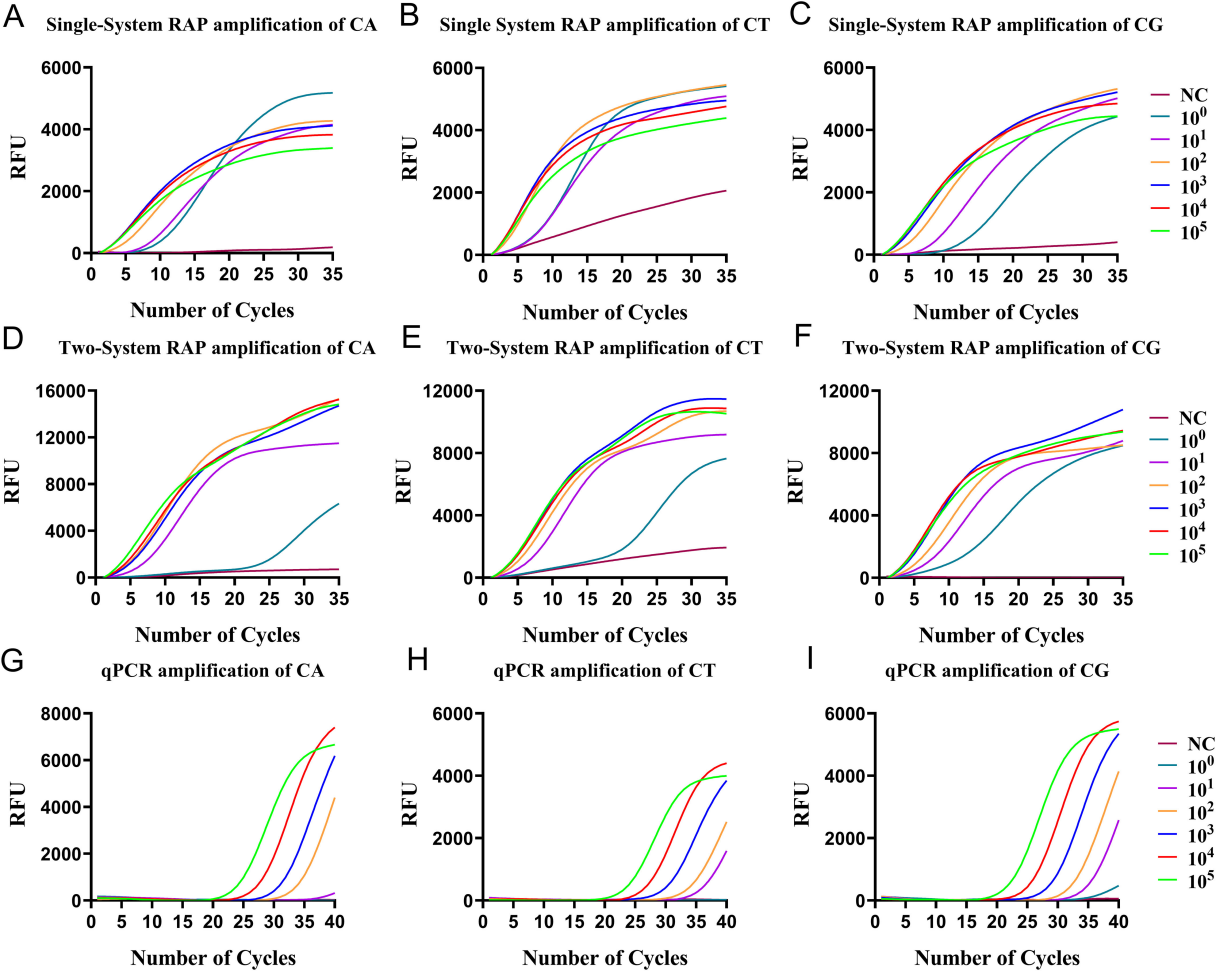
The sensitivity of single-system mRAP, two-system mRAP, and qPCR were analyzed using recombinant plasmids of 10^5^-10^0^ copies/μL. The CA **(A)**, CT **(B)**, and CG **(C)** recombinant plasmids were amplified by single-system mRAP; The CA **(D)**, CT **(E)**, and CG **(F)** recombinant plasmids were amplified by two-system mRAP method; The CA **(G)**, CT **(H)**, and CG **(I)** recombinant plasmids were amplified by PCR. NC, negative control; CA, *Candida albicans*; CT, *Candida tropicalis*; CG, *Candida glabrata.*.

The RAA and the qPCR system in the mRAP assay shared the same buffer system (single-system). Considering the optimal working magnesium ion concentration of the RAA reaction mixture and the qPCR reaction mixture are different, the optimal magnesium ion concentration working in both systems was determined to be 7mM by comparing the amplification efficiency of varying magnesium ion concentrations in single-system mRAP. Additionally, the ratio of different primers and probes of the targets in the mRAP system was adjusted to ensure that each target could be amplified with high efficiency. The optimal primer concentrations for targets CA, CT, and CG were found to be equal, while the probe concentrations were set at a ratio of 4:3:4. Finally, the RAA reaction time was adjusted from 12 min to 15 min to ensure the amplification efficiency of single-system mRAP. With these adjustments, the amplification of single-system mRAP could reach the plateau period in about 25 cycles, and the total detection time was about one hour. The sensitivity of the optimized single-system mRAP was comparable to that of the two-system mRAP, but the amplification curve was superior to the two-system mRAP when detecting low concentrations of plasmids (**Figures 2A–F**).

The disadvantage of single-system mRAP is that when using single-system mRAP for multiplexing detection, the negative control is not a straight line with unchanged fluorescence intensity, but a slightly sloped straight line with a slight increase in fluorescence height when using probes other than those labeled by FAM and VIC(HEX) fluorescence. However, the negative control can be distinguished from the positive amplification because the positive curve is similar to an “S-shaped” curve (**Figure 2B**).

### 3.2 Sensitivity, specificity and reproducibility of single-system mRAP assays

The sensitivity of single-system mRAP for the detection of CA, CT, and CG was evaluated using recombinant plasmids. A probabilistic analysis of the assay results revealed that the 95% detection limits for CA, CT, and CG were 4 copies/μL, 4 copies/μL, and 3 copies/μL, respectively. (**Figures 2A–C**). The results from eight replicate assays for each concentration of recombinant plasmids can be found in **Supplementary Table 1**. In comparison, the sensitivity of the qPCR method ranged from 10-1000 copies/μL (**Figures 2G–I**). The sensitivity evaluation indicated that single-system mRAP demonstrated a significantly higher detection rate for low plasmids concentrations than qPCR, particularly when the concentration was below 10 copies/μL.

The specificity assay in the bloodstream setting further demonstrated that the triple mRAP had good specificity, producing positive results only for CA, CT, and CG and no cross-reaction with 12 other common pathogens of bloodstream infections (**Table 2**). Additionally, no fluorescence amplification signals were observed in any of the negative controls, which further verified the specificity and reliability of the triple mRAP.

### 3.3 Evaluation of single-system mRAP and M1-mRAP in simulated blood samples

**Figure 3:**
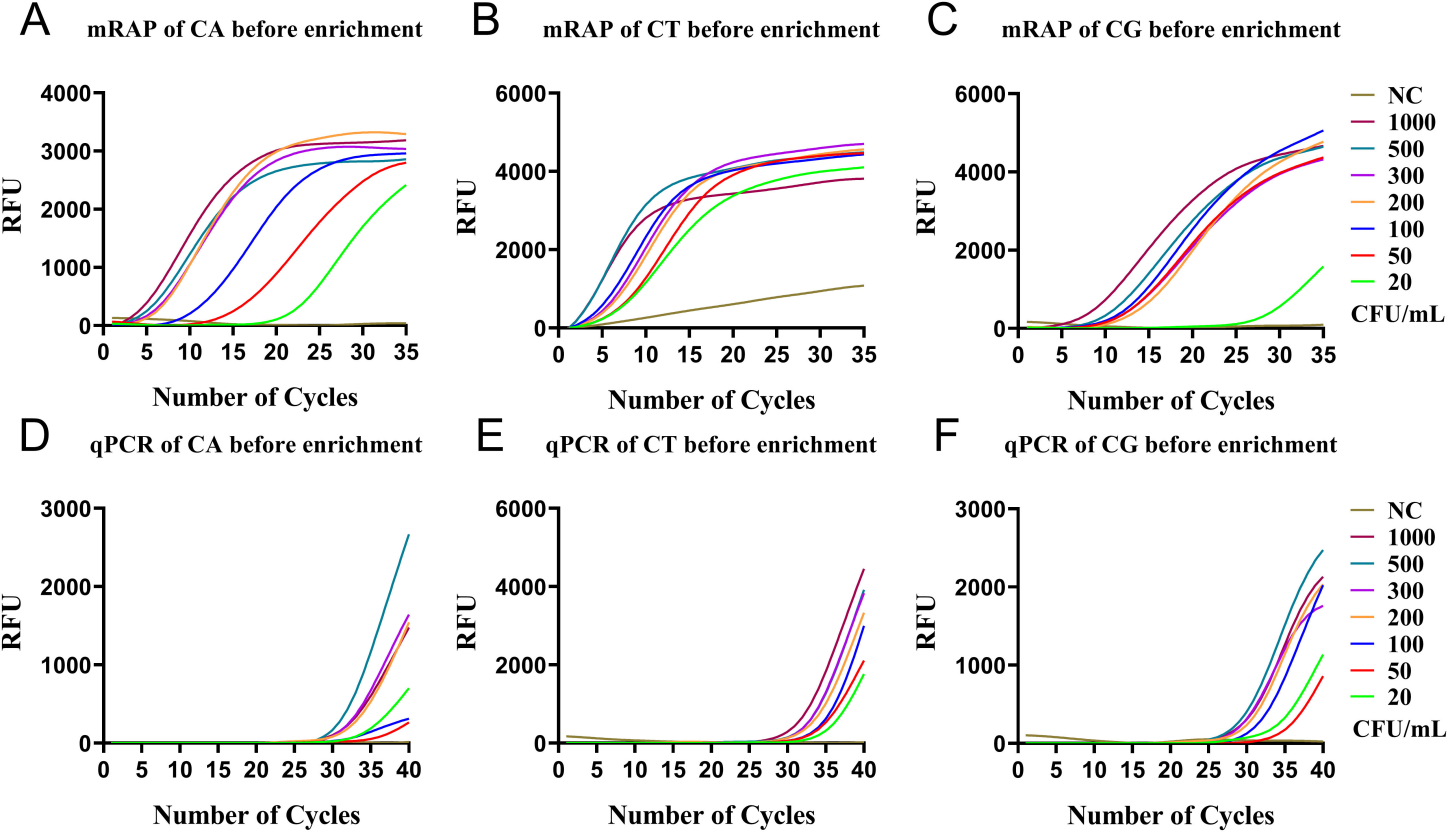
qPCR and single-system mRAP LODs assay for high-concentration simulated blood samples (20-1000 CFU/mL). The CA, CT, and CG mRAP assays **(A–C)**. The CA, CT, and CG qPCR assays **(D–F)**. CFU, colony forming unit; CA, *Candida albicans*; CT, *Candida tropicalis*; CG, *Candida glabrata.*.

Before the enrichment of M1 beads, the LOD of the single-system mRAP assay for CA, CT, and CG high-concentration blood simulated specimens (20-1000 CFU/mL) was 20, 20, and 50 CFU/mL, respectively (**Figures 3A–C**) and 200, 200, and 100 CFU/mL using qPCR, respectively (**Figures 3D–F**).

**Figure 4:**
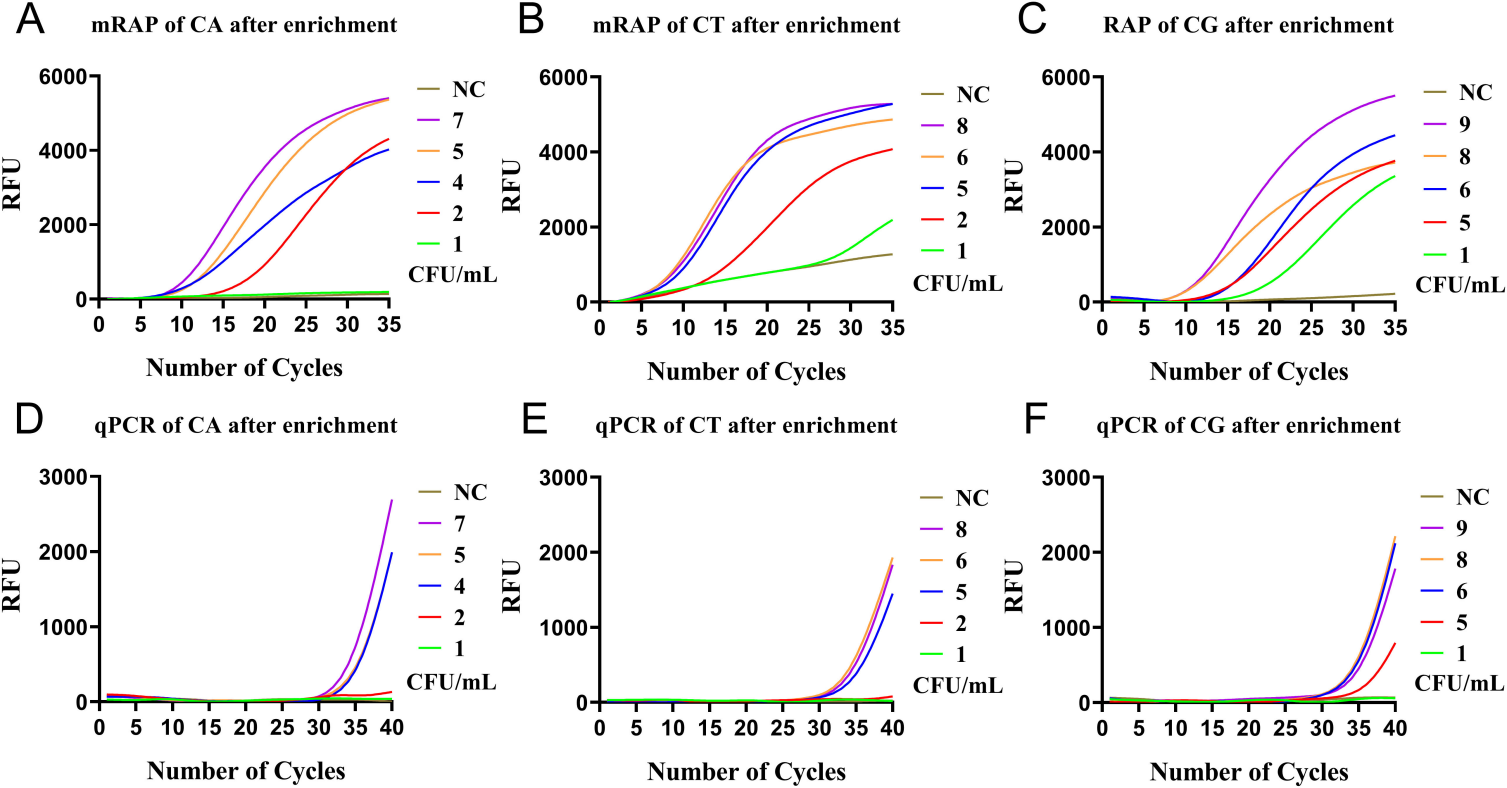
qPCR and single-system mRAP LODs assay based on M1-enriched low-concentration simulated blood samples (<10 CFU/mL). The CA, CT, and CG single-system mRAP assays **(A–C)**. The CA, CT, and CG qPCR assays **(D–F)**. CFU, colony forming unit; CA, *Candida albicans*; CT, *Candida tropicalis*; CG, *Candida glabrata.*.

After the enrichment of M1 beads, the LOD of single-system mRAP assay for CA, CT, and CG low-concentration blood simulated specimens (1-10 CFU/mL) were 2,2, and 1 CFU/mL, respectively (**Figures 4A–C**) and 4, 5, and 6 CFU/mL using M1-qPCR, respectively (**Figures 4D–F**). The calculated concentration of the low-concentration *Candida* simulation samples corresponded to the actual number of colonies on the plates (**Supplementary Figure 1**).

### 3.4 Evaluation of single-system mRAP and M1-mRAP in clinical samples

There were 129 clinical samples, including 120 non-blood samples and 9 fresh blood samples. Retrospective and prospective tests were performed on 120 non-blood samples and 9 blood samples, respectively. First, retrospective single-system mRAP and qPCR testings were performed on 120 non-blood samples(90 culture-positive and 30 culture-negative) (**Table 3**). The result showed that 85 (70.8%) blood culture-positive samples were positive for both single-system mRAP and qPCR, and 5 (4.2%) blood culture-positive samples had undetectable qPCR but tested positive for single-system mRAP. The remaining (25%) blood culture-negative samples were negative for both qPCR and single-system mRAP. Second, A prospective assay of 9 blood samples from patients with clinically suspected fungal infections showed that 1 (11.1%) sample was sequenced as *Candida tropicalis*, but qPCR was in the grey zone (not detected), and M1-mRAP was detected positively (detected). The remaining (90.9%) samples were negative for both sequencing and M1-mRAP assay. The results of the total of 129 clinical samples based on the mRAP and qPCR methods are shown in **Table 3**, with a total of 6 samples with different results (5 retrospective tests and 1 prospective test). The single-system mRAP results of 129 samples were compared with the qPCR results, and the Kappa values were 0.978, 0.934, and 0.956, respectively, with a p-value of <0.05, which indicates that there is a high degree of consistency between the two methods based on mRAP and the qPCR method.

**Table 3:** Detection of CA, CT, and CG in clinical samples.

Candida	mRAP		qPCR		Agreement	Kappa
	Positive	Negative	Positive	Negative		
CA	30	99	29	100	97.8%	0.978
CT	31	98	28	101	95.5%	0.934
CG	30	99	28	101	95.5%	0.956

The number of positive and negative samples is the total number of mRAP-tested non-blood samples and M1-mRAP-tested blood samples. Of these, 90/120 and 85/120 cases of mRAP and qPCR were detected in non-blood samples; 1/9 and 0/9 cases of mRAP and qPCR were detected in blood samples. CA, *Candida albicans*; CT, *Candida tropicalis*; CG, *Candida glabrata.*

All negative controls, consisting of simulated PBS samples, simulated blood samples, or healthy human blood samples, gave negative results in the single-system mRAP runs.

## 4 Discussion

Rapid and accurate identification of *Candida* presents a major diagnostic challenge in clinical *Candida* bloodstream infections, particularly when the concentration of circulating *Candida* cells in the blood is often ≤1 CFU/mL at the onset of infection (Pfeiffer et al., 2011). Current blood cultures and most blood culture-based tests are not perfect, mainly because of the long turnaround time and relatively low detection rate (McCarty et al., 2021). In this study, we established a novel diagnostic method for *Candida* bloodstream infections that supports the rapid and highly sensitive detection of CA, CT, and CG in blood samples. We used a homemade M1 bead to enrich blood samples, followed by routine nucleic acid extraction and finally amplification using a new high-efficiency amplification method (single-system mRAP). The M1-mRAP method can obtain rapid results in about 3.5 hours, provided that the M1 beads are prepared in advance, whereas blood cultures are much slower and may take 2-4 days. The M1-mRAP method was used in CA, CT, and CG low-concentration simulated blood samples (1-10 CFU/mL) and showed detection limits of 2, 2, and 1 CFU/mL. These data underscore the clinical value of this method in the early detection of bloodstream infections in patients. More importantly, samples missed (or in the grey zone) by conventional qPCR could be detected by the M1-mRAP method, demonstrating the potential of this method for clinical application.

The DNA extraction method of *Candida* is crucial for detection sensitivity, especially in blood samples. Whole blood samples contain free DNA and intracellular *Candida* DNA, while plasma contains only free DNA. Logically, the whole blood DNA extraction method may be more suitable for the detection of *Candida* in the blood due to more DNA and a larger sample volume. However, highly sensitive detection of blood samples has been challenging due to the susceptibility of whole blood DNA to DNA loss during nucleic acid extraction and the high background genomic DNA in blood, which can affect the amplification of microorganisms (Bacconi et al., 2014; Scharf et al., 2020), which may explain the higher detection rates of plasma and serum than whole blood in some studies (Lau et al., 2010). As a result, several studies have been conducted to achieve higher detection sensitivity in whole blood samples, including improved nucleic acid extraction and purification steps to isolate human DNA or using more tolerant enzymes (Scharf et al., 2020). In this study, we added an M1 beads enrichment step before routine blood nucleic acid extraction to enrich *Candida* and discard red blood cells, white blood cells, and other interfering components. We constructed a human mannan-binding lectin (i.e., M1) with six carbohydrate recognition domains (CRDs) and coated it on the surface of magnetic beads to form M1 beads (**Table 4**). M1 beads recognize polysaccharide residues on the surface of a wide range of pathogens and have a high binding affinity for *Candida* cells. The M1 beads capture *Candida* cells in the blood, increasing the relative concentration of *Candida* while discarding, by magnetic separation, components of the blood other than *Candida* cells that may affect amplification efficiency. Using M1 beads, we can sensitively detect *Candida* DNA directly from whole blood samples without relying on blood cultures. The effect of M1 beads in enriching *Candida* in blood was confirmed in our previous study, where the binding of *Candida* and M1 magnetic beads was directly observed in scanning electron microscopy (Chen et al., 2020). In this study, we compared M1-qPCR with standard qPCR using simulated blood samples, and there was a substantial difference in detection sensitivity (CA, 4 vs. 200 CFU/mL), demonstrating the potent role of M1 enrichment in blood *Candida* detection. It is important to mention that when using the M1 enrichment method for sample enrichment, the blood sample must be fresh to ensure the number of live *Candida* cells in the blood. M1 beads adsorb viable *Candida* cells, as shown by the Sabouraud’s Medium. In addition, the mannan structure changes depending on the fungal cell morphology. Three fungal morphologies (yeast, hyphae, and pseudohyphae) have been reported in blood infections. In particular, *Candida albicans* and *Candida tropicalis* can switch between the three morphologies. Further experiments are needed to verify the enrichment of M1 beads against a single *Candida* morphology. Finally, M1 beads are prepared for use within 14 days only. The M1 beads can be prepared and stored in a 4°C refrigerator before clinical testing to save time throughout the testing process.

**Table 4:** Definitions of commonly used abbreviations.

Abbreviations	Definition
M1	recombinant human mannan-binding lectin
M1 protein	recombinant human mannan-binding lectin protein
M1 beads	Magnetic beads coated with M1 protein
RAA	recombinase-aided amplification
RAP	recombinase-aided PCR
mRAP	multiplex recombinase-aided PCR
CFU	colony forming unit
CA	*Candida albicans*
CT	*Candida tropicalis*
CG	*Candida glabrata*

A variety of PCR-based assays are widely used in the molecular diagnostic approach for *Candida*. However, variable analytical sensitivity and limited validation of these assays in a prospective setting make their utility for clinical testing uncertain (Pham et al., 2024). Recently, the RAA/RPA method has been widely used in the detection of infectious diseases due to its high sensitivity and short detection time (James and Macdonald, 2015). However, the complexity of RAA fluorescent probes has limited its widespread application. Therefore, in this study, we developed a single-system mRAP method that is more sensitive than conventional qPCR or RAA alone. The single-system mRAP method bypasses the use of fluorescent probes in RAA and instead uses a simple and easy-to-design qPCR probe as the fluorescence detection probe. The single-system mRAP takes advantage of the rapidity and sensitivity of the RAA method and the flexibility of the Taqman probe of the qPCR method to achieve rapid and highly sensitive detection of pathogens while avoiding the use of RAA probes, which are challenging to design. The previously established two-system RAP has demonstrated its clinical use in *Candida albicans* and *Candida tropicalis* (Zhang et al., 2023). Subsequent optimization of the system in this study led to the establishment of a single-system mRAP for the simultaneous detection of the three *Candida* species without cross-reactivity. The single-system mRAP has good analytical sensitivity and high specificity for the detection of 4, 4, and 3 copies/μL of CA, CT, and CG standard DNAs. Clinical testing of 120 diagnosed non-blood samples using the single-system mRAP method showed agreement between single-system mRAP and blood culture results. The results of single-system mRAP and qPCR showed mostly agreement except for 5 cases of qPCR undetermined but were detected as positive by single-system mRAP, demonstrating that the single-system mRAP method is a potential method for detecting low-concentration clinical samples that cannot be identified by conventional methods. Meanwhile, two samples from patients with dual *Candida albicans* and *Candida tropicalis* infections were accurately detected by single-system mRAP, demonstrating the ability of single-system mRAP to be used to detect multiple *Candida* infections accurately.

When the proposed workflow of M1-mRAP is compared to two other excellent platforms for the detection of *Candida* blood samples: the T2 *Candida* panel (FDA-approved) and the LightCycler SeptiFast (Conformité européenne approved) system (Camp et al., 2024), the M1-mRAP method has similar sensitivity to T2 *Candida* panel(1-2 vs. 1-3 CFU/mL), but the M1-RAP method is simpler and less expensive. In addition, M1-mRAP can be amplified using conventional qPCR instruments without relying on unique instrumentation, making it suitable for use in most laboratories. On the other hand, M1-mRAP had better CA, CT, and CG detection sensitivity than the LightCycler SeptiFast system (2 vs. 30 CFU/mL; 2 vs. 30 CFU/mL; 1 vs. 100 CFU/mL) and shorter detection times (3.5 vs 6-7 hours). The advantages of shorter assay duration and high sensitivity of M1-mRAP also demonstrated its potential clinical applicability despite the detection of only three *Candida* species. Finally, we used the M1-RAP method to detect 9 fresh blood samples from patients with prospectively suspected fungal bloodstream infections. Only one positive sample confirmed by mass spectrometry was not detectable by qPCR but positive by M1-mRAP. Although the high sensitivity of M1-mRAP was not fully demonstrated, it offers evidence that the M1-mRAP assay can be applied to the detection of blood samples from patients with *Candida* infections.

However, the M1-mRAP assay has several limitations. The first is that the M1-mRAP is currently only qualitative and not quantitative, probably due to the fact that the first stage of the RAA reaction has increased template amplification to the qPCR detection threshold. Secondly, Only five common *Candida* species were included in the specificity assay, and other rare *Candida* species were not included in the specificity assay due to limited experimental conditions. Due to sequence similarity, the primers and probes in this study partially cross the sequences of some *Candida* species, such as C. dubliniensis, C. bracarensis, and C. nivariensis. However, this does not affect the medication treatment of patients with *Candida* infections because the onset of *Candida* other than the five species is rare. Another reason is that the Infectious Diseases Society of America (IDSA) recommends echinocandins as an initial treatment for all adult patients with candidemia (Gold et al., 2021). In future studies, we will closely monitor medication changes for rare *Candida* and collect additional *Candida* strains for specificity assay. Finally, more clinical samples, especially fresh whole blood samples with single or multiple *Candida* infections, should be validated for assay performance using M1-mRAP. Our next steps will be to integrate the multiplex RAP with a POCT device, develop a small, fully automated device for on-site testing, and apply it to a wider range of bloodstream infection pathogen types (Ma, 2022).

## Data Availability

The original contributions presented in the study are included in the article/**Supplementary Material**, further inquiries can be directed to the corresponding author/s.
