# Supplementary material for: Rapid and sensitive identification of Candida in blood based on M1 beads enrichment combined with multiple recombinase-aided PCR: a culture-independent approach
Source: Front Cell Infect Microbiol. 2025 Mar 13;15:1552529. doi: 10.3389/fcimb.2025.1552529 (PMC11966459; doi:10.3389/fcimb.2025.1552529)
Supplement: Supplementary file 1 [file SupplementaryFile1.docx]

Supplementary Material

# Supplementary Figure and Table

## Supplementary Figure

**
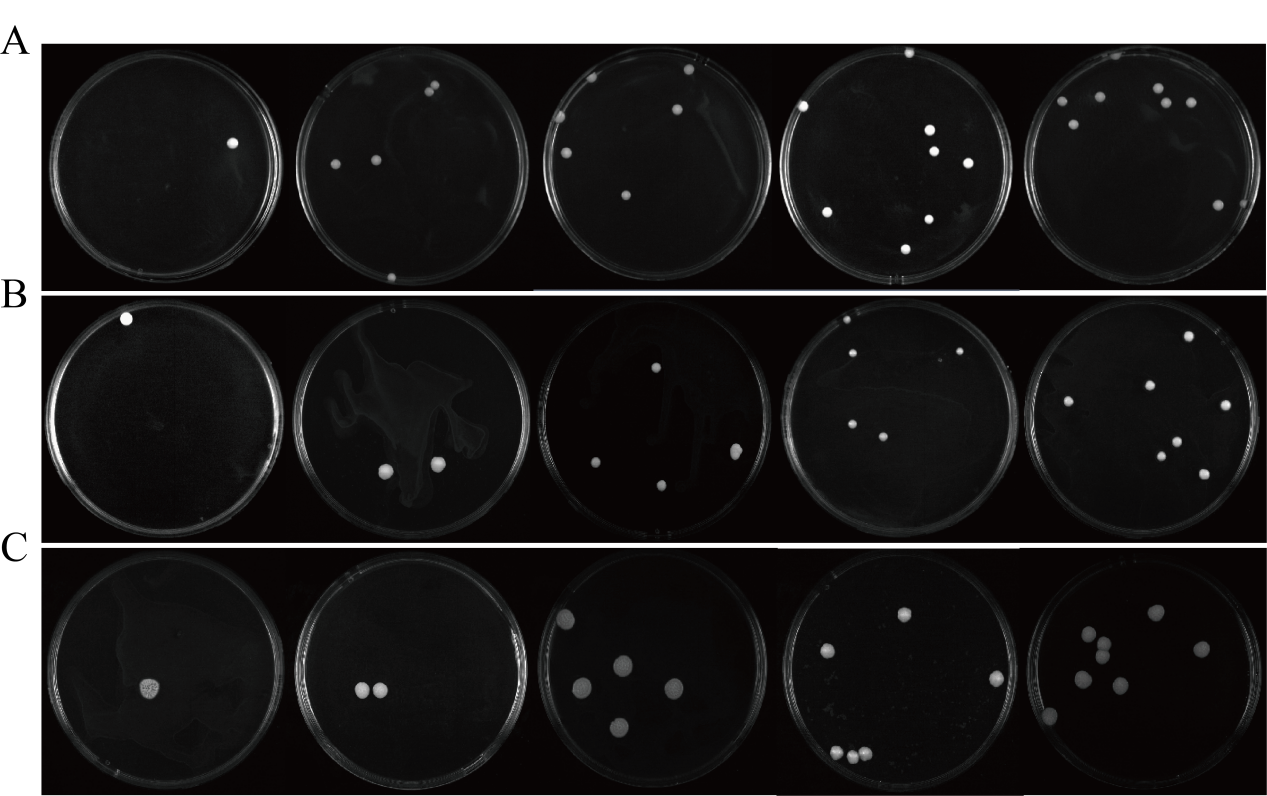
**

**Supplementary Figure 1.** Plate growth of low-concentration simulated specimens enriched by M1 beads. A, Simulated blood specimens with *Candida glabrata* concentrations <10; B, Simulated blood specimens with *Candida albicans* concentrations <10; C, Simulated blood specimens with *Candida tropicalis* concentrations <10

## Supplementary Table

**Supplementary Table 1** The reproducibility of single-system mRAP

| Standard DNA(copies/reaction) | CA | CT | CG |
| --- | --- | --- | --- |
| 10^5^ | 8/8^a^ | 8/8 | 8/8 |
| 10^4^ | 8/8 | 8/8 | 8/8 |
| 10^3^ | 8/8 | 8/8 | 8/8 |
| 10^2^ | 8/8 | 8/8 | 8/8 |
| 10^1^ | 8/8 | 8/8 | 8/8 |
| 10^0^ | 2/8 | 4/8 | 4/8 |

Note: ^a^The first number indicates the number of times a positive result occurred. The second number indicates the experiment was repeated 8 times.
